# The Gift: Ethically Indicated Euthanasia in Companion Animal Practice

**DOI:** 10.3390/vetsci8080141

**Published:** 2021-07-24

**Authors:** Anne Quain

**Affiliations:** Sydney School of Veterinary Science, Faculty of Science, University of Sydney, Camperdown 2006, Australia; anne.quain@sydney.edu.au

**Keywords:** euthanasia, companion animal, veterinarian

## Abstract

The majority of companion animals seen by veterinary practitioners will die by euthanasia. Yet euthanasia can be a source of moral stress for veterinary team members, even when ethically indicated. In this discussion, I explore when euthanasia is ethically indicated and discuss the potential impact of ethically indicated euthanasia on veterinary team members. In particular, I challenge the analogy that the veterinarian performing ethically indicated euthanasia is akin to an executioner, arguing that this analogy is both inappropriate and potentially harmful. Finally, I discuss how we can support ourselves and our colleagues in relation to euthanasia, so we can attend to and maximise the welfare of our patients at the end of their lives.

## 1. Introduction

**Figure 1:**
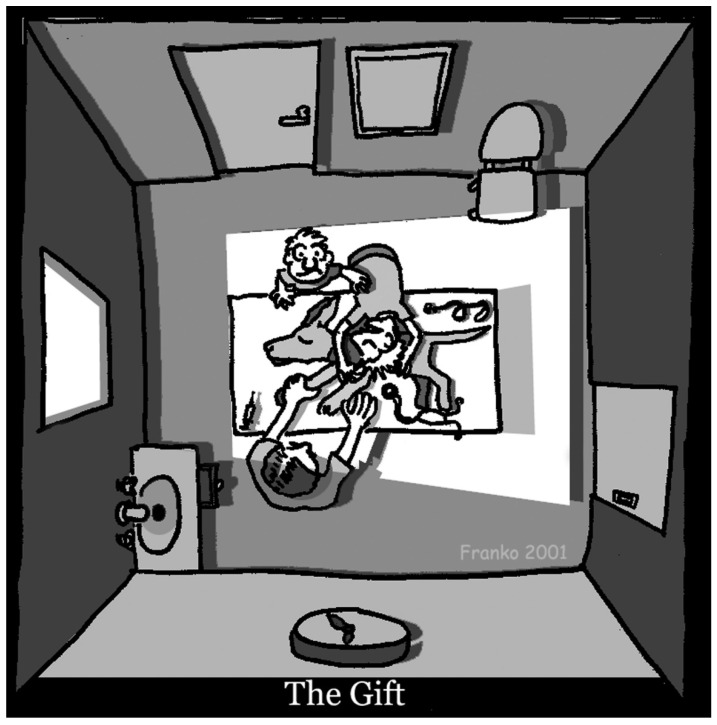
“The Gift” by veterinarian and animator Frank Gaschk.

“The Gift”, a cartoon by veterinarian and illustrator Frank Gaschk (Figure 1), depicts the immediate aftermath of the euthanasia of a beloved pet dog. The dog lies peacefully on the table in the consulting room. The dog’s lead is curled up on the table, no longer needed. On one side of the table, a heartbroken owner is slumped over the body of the dog. Beside them, a person stands, one hand offering comfort to the other grief-stricken party as they look upwards, perhaps in a vain attempt to hold back tears, perhaps looking to a higher order for some sort of solace. On the other side of the table is the veterinarian, whose face we cannot see. One hand appears to be holding off the cephalic vein, the syringe now resting empty on the table.

The title of the image, “The Gift”, refers to the privilege we have as veterinarians to end suffering. As it turns out, that gift is a double-edged sword.

When I first saw this image, it hit me. As a lifelong owner, or guardian, of companion animals, I can relate to that visceral experience of loss. The enormity of seeing an animal’s final breath, crying tears into the fur of a body that will never be reanimated. But as a veterinarian, I know exactly what it is like to exercise that gift.

The artist’s naïve depiction readily flickers to life in my imagination. After auscultating the dog’s chest and confirming the absence of a palpebral reflex, I declare softly: “she’s gone”. The owner, who it seems has been holding his breath until this moment, audibly sobs, folding himself over the dog, feeling the warmth still in her body. We are both relieved that the adverse euthanasia events I warned about-agonal gasping, vocalization-did not occur.

There is silence in the room, except for the occasional sniffle. I pass the client a box of tissues and tell him that I am so sorry. And I am. As he wipes his eyes, he says to me “this must be the hardest part of your job”.

How do I respond? The truth is, performing ethically indicated euthanasia is not the hardest part of my job. My mind swirls with potential alternative candidates. Being in proximity to suffering (animal or human). Interpersonal conflict. Decision-making in the face of incomplete information. Causing unintended harm. Unexpected complications. The difficulties of addressing animal welfare compromise on a broader scale. Triage. Time management. Attending to self-care. Trying to comprehend the global loss of biodiversity and why some animals receive veterinary care while the vast biomass of animals on the planet will not.

Performing ethically indicated euthanasia does not feature on the list that springs immediately to mind because I struggle more with these other job demands and because it alleviates animal suffering. Ethically indicated euthanasia is the work of a compassionate clinician, not an executioner.

And yet, anecdotally, some veterinary team members feel like executioners when they perform ethically indicated euthanasia. Indeed, the 2021 Mars Society of Veterinary Medical Ethics student essay topic stated that “even when animal euthanasia is ethically indicated, many veterinarians and vet staff become stressed because they feel like executioners” [1].

In this discussion, I will talk about when euthanasia is ethically indicated and the impact of ethically indicated euthanasia on veterinary team members. I will explore whether a veterinarian who performs ethically indicated euthanasia is really akin to an executioner and how we may be able to support ourselves and our colleagues in relation to euthanasia so that we can attend to the welfare of animals.

## 2. When Is Euthanasia Ethically Indicated?

There are differences of opinion about when euthanasia—derived from the Greek *eu* for good and *thanatos* for death—is ethically indicated [2].

The American Veterinary Medical Association (AVMA) Guidelines for the Euthanasia of Animals describe two co-dependent, necessary conditions for euthanasia, notably:

1. “humane disposition [of the veterinarian] to induce death in a manner that is in accord with an animal’s interest and/or because it is a matter of welfare;”
2. “the use of humane techniques to induce the most rapid and painless and distress-free death possible” [3].

The late animal rights philosopher Tom Regan proposed stricter criteria for ethically-indicated euthanasia, notably:

1. Killing must be by the most painless means possible;
2. That it must be believed to be in the animal’s best interests, and this must be a true belief;
3. One who kills must be motivated by concern for the interest, good or welfare of the animal involved [4].

Unlike the AVMA Guidelines, Regan’s criteria preclude the use of the term “euthanasia” to describe the humane killing of healthy animals. His criteria imply that the impact of euthanasia on animal welfare should be positive (unless we are genuinely misled about the animal’s interests) in that it eliminates actual or potential suffering. While the focus of this discussion is companion animals, it is worth noting that Regan’s criteria preclude the slaughter of animals, for example, for food, because this is performed against the interests of an animal, as a subject of a life.

Another approach that may be applied to decision-making is the UK Farm Animal Welfare Council’s quality of life assessment, by which animals are determined to be leading “a life worth living”; “a life not worth living” and “a good life” [5]. Application of this criteria to the euthanasia decision requires balancing and weighing up factors and experiences that lead to negative and positive welfare. According to this approach, euthanasia would be indicated if overall an animal’s life is, or is immediately about to become, “not worth living”.

But quality of life is challenging to assess objectively. Who decides, and importantly, how is that decision made? Unlike medical euthanasia, veterinary euthanasia—like all veterinary procedures-is carried out without the consent of the patient, or direct knowledge of the patient’s interests. Different stakeholders (for example, the veterinarian and the owner of the animal, the veterinarian and colleagues, or two different owners of the same animal) may have different views about an animal’s quality of life, prognosis and interests. According to the AVMA, “what constitutes a good life and what counts as an impoverished life, or one that has limited quality such that the death of the animal is the most humane option, are research areas in need of further study by the veterinary and ethics communities” [3].

In an attempt to aid decision-making, the latest AVMA Euthanasia Guidelines incorporate both a decision tree to help veterinarians decide whether euthanasia is warranted in situations where this is not clear, and a decision matrix to evaluate the morality of their decisions in relation to patient euthanasia. They prompt the user to consider alternatives (“can it be rehomed?”) and the decision itself (“Am I comfortable with my decision? If there is something that troubles me, what is it?”) [3].

Indeed, the addition of these decision-making tools to the AVMA Euthanasia Guidelines is contextualised as potentially assisting “veterinarians who may be struggling with what may seem to be gratuitous euthanasia, the acceptability of certain procedures, and the sometimes routine nature of performing euthanasia” [3].

While these tools may be helpful, they do not provide answers. Like all ethical frameworks, they ultimately rely on the professional and personal judgement of the decision-maker. What constitutes ethically indicated veterinary euthanasia is by no means clear-cut.

Ethically indicated euthanasia may be better defined by what some argue that it *is not.* Humane killing that is not in the best interests of the individual animal may include but is by no means limited to, convenience euthanasia, economic euthanasia and objectionable euthanasia.

Convenience euthanasia has been defined as “euthanasia…based on human needs, such as economics, changes in personal circumstance, lack of time, capacity or desire to care for the animal, rather than animal welfare” [6] or “euthanasia of a physically and psychologically healthy animal” [7,8]. It has also been referred to as “non-justified animal euthanasia”, that is, where an alternative to euthanasia is available but declined by the owner [9,10] or potentially not explicitly offered by the veterinarian.

Economic euthanasia has been defined as “a condition in which euthanasia is elected based primarily, principally, or to a large degree on the cost of veterinary medical care; a condition in which veterinary care is bypassed based on the anticipated cost of care, and the progression of illness leads to euthanasia; or a condition in which veterinary care is sought and minimal or no testing/treatment is elected based on the costs of care, resulting in eventual euthanasia” [11].

Objectionable euthanasia has been defined as “euthanasia that the veterinarian disagreed with” [12]. Veterinary team members may refuse to perform or assist in euthanasia that they find objectionable, but this is not always the case. A survey of UK veterinarians (*n* = 58) found that 81 per cent had refused to perform euthanasia, with the median frequency of euthanasia refusal “yearly or less” [13]. In the same study, 71 per cent of respondents reported wanting to refuse a request but performing euthanasia anyway, with a median frequency of “yearly or less.” Veterinarians varied in their reasons for refusing euthanasia and for wanting to refuse euthanasia. The most common reasons for euthanasia refusal were lack of a perceived legitimate reason for euthanasia (for example, a young healthy animal), better options available (for example, the animal was rehomeable), owner decision making (for example, lack of clarity around consent), insufficient owner interests (for example, convenience or economic reasons cited) or a perception that the reason for euthanasia was the owner’s fault (for example, the problem was caused by the owner, the owner had not taken steps to rectify the problem). The most common reasons for performing euthanasia in these cases were concerns about what would happen to the animal otherwise, lack of alternative options, the owner’s interests (including financial limitations and mental state), pressure from a client, employer, colleagues or the expectation that the client would request euthanasia by another vet), and broader implications (for example, refusing to perform euthanasia could deter other clients from seeking veterinary assistance when needed). In a survey of USA veterinarians (*n* = 484), 80 per cent reported declining a euthanasia request at some point in their career (52 per cent reported a frequency of once every few years, 32 per cent reported a frequency of at least a few times a year). Common reasons for euthanasia refusal were concern that a client may seek alternatives that worsened animal welfare, difficulty declining a request when the client had made a decision, and concerns about jeopardizing client relations [6]. In a survey of North American veterinarians (*n* = 889), the majority (*n* = 776; 93.05%) had received what they considered to be an inappropriate request for euthanasia, although 63.67% (*n* = 531) reported that this was rare [14]. Of these, respondents never (*n* = 308, 39.74%) or rarely (*n* = 292, 37.68%) complied with these requests. Finally, a study of 183 veterinary anaesthetists found that 4% (*n* = 9) raised euthanasia of healthy animals or animals with diseases they perceived to be readily treatable as concerns [15].

The animal welfare outcomes of euthanasia refusal by veterinary team members have not been investigated. “Inappropriate” requests to perform euthanasia on animals were associated in variable stress levels among veterinarians in one study, causing mild distress (*n* = 253, 32.81%), moderate distress (*n* = 344, 44.62%) or severe distress (*n* = 144, 18.68%) [14].

There is significant potential for disagreement about what constitutes convenience, economic and objectionable euthanasia, and a comprehensive analysis is beyond the scope of this paper. For the purposes of this discussion, I will consider ethically indicated euthanasia to refer to euthanasia performed in what are believed to be the animal’s interests and which is not considered to be *primarily* motivated by convenience, economics or reasons to which veterinary team members object.

## 3. What Is the Impact of Ethically Indicated Euthanasia on Veterinary Team Members?

Recently there has been increased discussion about the psychological wellbeing of veterinary team members, prompted by reports of high suicide rates among veterinarians [16,17,18,19]. Some authors speculate that one potential reason for this higher risk was veterinarians’ tolerance to the process of effecting a peaceful death due to routine performance of euthanasia [16].

In the 1980s, philosopher Bernard Rollin introduced the term “moral stress” in the veterinary literature to describe the impact of the killing of healthy animals on shelter staff. He wrote that moral stress “is encountered by those whose jobs require that they kill animals for reasons other than the alleviation of intractable pain and suffering; i.e., for reasons that are not to the direct benefit of the animal.” [20]. He has persistently maintained that euthanasia, if performed for reasons other than in the animal’s interests, will negatively impact those charged with the task [21].

Compassion fatigue has been defined as a form of secondary traumatic stress experienced by those who care for humans or animals who experience suffering [22]. Studies of the impact of euthanasia on shelter staff have found a correlation between performing euthanasia and compassion fatigue [23,24,25].

However, as discussed in the previous section, the humane killing of animals for reasons not in the interests of these animals is not the same as ethically indicated euthanasia, and the use of the term “euthanasia” in these contexts may not be appropriate [2]. The impact of performing ethically indicated euthanasia may therefore be different. In a study of South Australian veterinarians working with any species of companion animal (*n* = 103), almost 40 per cent had “high” or “very high” scores on the Compassion Fatigue Short Scale (CFSS), yet there was no significant correlation between CFSS scores and frequency of euthanasia [26].

In the study of Australian veterinarians by Crane and colleagues, while performing euthanasia, in general, was the most frequently reported stressor, it was the least morally significant stressor [27]. Performing euthanasia for reasons the veterinarian did not agree with was less common but a more morally significant stressor. Furthermore, performing euthanasia may have moderated the impact of depression on suicide risk in veterinarians [12]. It may be that euthanasia, ethically indicated and performed well, is a source of compassion satisfaction [28]—the ability to take pleasure in work, specifically through helping others.

Failure to perform euthanasia when ethically indicated is a reported stressor among veterinary team members. In a study of UK veterinarians (*n* = 58), clients wishing to pursue treatment despite poor animal welfare or poor quality of life was rated as the most stressful ethical challenge, more stressful than a request to euthanase a healthy animal [29]. In a study of North American veterinarians (*n* = 889), 56.95% sometimes and 21.58% often managed cases where they felt an owner requested treatment which they perceived to be futile [14], while 18% of veterinary anaesthetists felt that euthanasia was delayed beyond the point that they felt was appropriate, and 14% facilitated procedures that they felt did not improve an animal’s quality of life [15].

A qualitative study based on focus groups and individual interviews of veterinarians in Ontario found that participants reported improved personal wellbeing when they perceived that they had effected a “good death” [30]. For most participants in this study, facilitating a “good death” was framed as a positive act and a means of ending patient suffering. It was also seen as supporting the wellbeing of the client. Conversely, when participants did not feel that they facilitated a good death, for example, unwanted side-effects or a perceived delayed decision to euthanase by the owner, they experienced reduced wellbeing, reduced job satisfaction and a perception that the client was negatively impacted.

Ethically indicated euthanasia may be a stressor because of pressure to be technically competent under the observation of distraught owners. Intravenous access may be difficult to establish due to the condition of the animal [31]. Adverse euthanasia events are common, occurring in 26.5 to 52 per cent of canine euthanasias in clinical practice [32,33]. Common adverse euthanasia events include changes in respiratory rate and character, agonal gasping, ptyalism, emesis, vocalization, involuntary muscle activity (including myoclonus, muscle fasiculations or tremors), opisthotonos, urination or defecation, and intravenous catheter-related complications [33]. Even expected perimortem events may be distressing to clients or inexperienced team members witnessing euthanasia, increasing pressure on the veterinary team to anticipate and carefully manage such events [34]. It is important to appreciate that performing ethically indicated euthanasia does not preclude moral stress. Our training gears us towards saving lives, yet at a point that can be hard to define, we pivot and end a life that we may have worked hard to save. This may cause doubts: Have we failed? Are we, or the owners, being influenced by factors that we may not feel should influence us, such as practical or financial constraints? If we are committed to acting in the patient’s interests, should we consider the client’s interests at all? Are we sure euthanasia is ethically indicated? We need to clarify the sources of our moral stress [35].

Ethically indicated euthanasia is also a source of grief for veterinary team members. It has been estimated that veterinarians experience the loss of a patient around five times more frequently than general medical practitioners [36]. Grief over patient loss [37], or vicarious grief emerging from witnessing clients’ loss, rather than moral stress, may explain some of the impact of ethically indicated euthanasia on veterinary team members.

Euthanasia, including ethically indicated euthanasia, involves emotional labor for veterinary team members, who must manage their own emotions while supporting the client [26,34]. In a study of South Australian veterinarians (*n* = 103), 40 per cent of respondents reported that their mental and/or physical health were impacted by interactions with clients grieving the loss of a companion animal [26].

One of the challenges in determining the true impact of ethically indicated euthanasia on veterinary team members is that the impact of euthanasia may be confounded by many potential job demands that may be experienced concurrently [38].

## 4. Is a Veterinarian Who Performs Ethically Indicated Euthanasia Really Akin to an Executioner?

It may be first useful to ask, is a veterinarian or veterinary team member performing euthanasia, ever akin to an executioner? Historically, the analogy may have been appropriate.

In the Middle Ages, executioners carried out stray animal control among their duties, playing a role that was later fulfilled by veterinarians [39]. In addition to performing executions, executioners played an important role in removing stray and deceased animals from towns and neighbourhoods, as well as doing other unpleasant work, including cleaning sewage systems and wells. According to Chrószcz and colleagues, the archaeozoological record of this period shows that “the human–animal relationship was far from the humane treatment typical for pet animals” [39]. Executioners killed animals largely as a means of population control rather than with any consideration of the animals’ interests.

Insofar as veterinarians and shelter staff are involved in the destruction of animals for reasons including population control or public health, it could be argued that they—like executioners—engage in society’s “dirty work”, that is work that is perceived as “disgusting”, “degrading” or “objectionable”. Engaging in dirty work poses a threat to one’s identity and has potentially harmful effects on an individual’s wellbeing [40].

While there are no published studies suggesting that veterinarians and staff feel like executioners, a survey of 177 veterinary clients in Ontario reported that 16 per cent agreed with the statement that “I felt like a murderer having my pet euthanased” [41]. Without additional information, it is difficult to know why these owners felt that this analogy was appropriate. Was it simply because consenting to euthanasia was understood as an act of commission akin to pulling a trigger? Was it because they felt that euthanasia was not, in fact, in their animal’s interests? Was the animal’s death distressing? Or was the selection of this response to a survey question a manifestation of grief?

It is possible that in situations, which for the purposes of this discussion do not meet the criteria for ethically indicated euthanasia, that veterinary team members involved may feel akin to executioners. However, the analogy is problematic, and failure to challenge this could lead to unintended consequences. These might include a negative impact of performing ethically indicated euthanasia on veterinary team members and even avoidance of euthanasia by these individuals, which may prolong animal suffering. It may lead to a negative self-perception. On the other hand, challenging this analogy may improve self-perception and may ultimately allow veterinary team members to reframe euthanasia as a tool in preventing suffering.

Execution is heavily contested and is not carried out in all jurisdictions [42]. In contrast, veterinary euthanasia is an almost universally legal, socially accepted means of ending suffering. In retrospective studies of the records of deceased companion animals (*n* = 130 cats, 68 dogs, and N = 26,986 dogs, respectively), only 8.3–9 per cent had an unassisted death [43,44].

An executioner has the role of taking human life in effecting a legally sanctioned death penalty imposed as punishment for a crime [45]. Veterinary euthanasia is not punitive. Rather, it is performed primarily to prevent actual or potential animal suffering.

Methods of execution may lead to suffering. In fact, the AVMA Euthanasia Guidelines were contrasted with methods of execution by lethal injection to highlight that the methods of lethal injection anaesthesia in executing prisoners were cruel [46]. While the AVMA raised concerns about this comparison [47], the implication was, in fact, that companion animal euthanasia was a much more humane process, with more concern shown for animal welfare in veterinary euthanasia than is shown to prisoners executed by lethal injection.

Executioners function as technicians who carry out their actions on behalf of society via the legal system. In contrast, veterinarians typically have a role in advising the client around euthanasia decision-making, through which they can advocate for their patients. Furthermore, veterinary team members increasingly engage in shared decision-making, such that ethically indicated euthanasia is the result of informed discussion between the veterinary team and the owner [48].

Finally, there are many who would argue that the world would be better without capital punishment. In contrast, veterinary team members report refusal of euthanasia recommendations by a client, or requests for futile care despite a poor quality of life, as among the most stressful ethical challenges encountered in companion animal practice [6,15,29].

It may be useful for veterinary team members to engage in a thought experiment, imagining a world in which veterinary euthanasia does not occur. In this world, a high proportion of companion animals would be at risk of experiencing protracted and potentially extreme suffering, with compromised quality of life. This would lead to moral stress in veterinary team members, who may feel that their skills and resources are being used to facilitate compromised animal welfare.

## 5. How Can We Support Colleagues in Regard to Ethically Indicated Euthanasia?

Veterinary team members are generally not trained in human psychology and are not in a position to diagnose or treat themselves or colleagues for mental illness, including depression. However, our behaviour impacts the wellbeing of those around us [49,50]. We know that veterinary team members suffer from high rates of psychological distress, including depression [51,52,53]. We also know that euthanasia is an important means of eliminating animal suffering in veterinary settings and may be unavoidable. It is possible that the equation of ethically challenging euthanasia with execution is a sign, rather than a cause, of depression. Similarly, ethically indicated euthanasia may be stressful not because it makes a veterinary team member feel like an executioner but because it is associated with grief. The enormity of loss may overwhelm a sense that we are otherwise doing good.

Veterinary social work services are offered in some settings, in particular, veterinary teaching hospitals [54]; however, they are rarely available to veterinarians working in private practice. Professional associations and workplaces should ensure that counselling is available, for example, through employee assistance programs, to provide support. A survey of veterinary health professionals (*n* = 98) who accessed the Vetlife Health Support (VHS) telephone service found that 97 per cent had a positive experience, and 98 per cent felt respected and listened to [55]. While the study did not determine whether the use of the service led to a positive mental health outcome, overall, respondents reported a significant improvement in relationships with others and were more likely to access formal mental health care services following contact with VHS.

Help-seeking should be role-modelled and normalised in workplaces, and structural barriers to help-seeking removed. For example, it has been suggested that employees be permitted to schedule meetings with mental health professionals, such as veterinary social workers or counsellors, in work hours if required [56]. Mental health professionals are well-practiced at challenging negative thoughts and may be able to help veterinary team members clarify the source of their distress. Some veterinary team members may have limited capacity for dealing with euthanasia, even when ethically indicated. It may be possible for their exposure to euthanasia to be minimised for periods of time.

We can create psychologically safe spaces in which we can discuss, without judgement or criticism, concerns about euthanasia. Questioning the ethical basis for decision-making and considering potential alternatives are important in ensuring that euthanasia is performed for appropriate reasons and not just “because it is always done this way”. We need to get into the practice of articulating and sharing our ethical justifications for decisions, including euthanasia, clarifying our reasons for potential discomfort [35]. It is important that veterinary team members feel that their concerns are acknowledged by others, even if those concerns are not shared [15]. It is important to focus not just on the costs of euthanasia but the benefits.

Discussion can lend perspective: every living being will stop living, at some point, whether medically assisted or not. No single veterinary team member can change that fact. Our job is not to cheat mortality on behalf of our patients but to work with their owners to ensure their quality of life is maximised to the best of our ability. We cannot avoid death, but we can facilitate a good death.

We can accept that euthanasia is complex and that death when it represents the end of suffering, can be a positive, while sad, experience for veterinarians and animal owners [30]. We can share our positive experiences of euthanasia with colleagues and recraft our perspective about work by positively reframing ethically indicated euthanasia [38].

We can refine our techniques to ensure that we achieve the most humane death possible and minimise side-effects (for example, reaction to injection or excitation) [57] which may lead to distress in animal patients, their owners, and the veterinary team members. For example, sedation of animals prior to euthanasia facilitates minimal restraint of animals and may relieve fear, anxiety and stress [58].

We can work on improving the euthanasia experience for veterinary team members. This may involve developing communication skills and decision-making around euthanasia and engaging in continuing professional development. Matte and colleagues found that veterinarians struggled more with navigating the euthanasia decision-making process, particularly if this required multiple or prolonged consultations [30]. They speculate that the involvement of a veterinary social worker or other professional, such as a counsellor, may reduce this source of stress.

At times our stress or grief may cause us to forget our reasons for performing euthanasia. Through preventing the suffering of animals and having the time, resources and skills to facilitate a good death, it can provide opportunities for self-actualisation and be a source of meaning, which in turn may improve the wellbeing of veterinary team members [38].

## 6. Conclusions

According to Persson, “euthanasia does present a unique option for veterinarians to bring relief to both the patient and the owner in many cases and leaves the veterinarian with a powerful tool that physicians in human medicine are missing” [35].

If we reflect on the sources of moral distress, if we can share our thoughts and feelings with colleagues, and if we can give ourselves space to experience grief as well as satisfaction on facilitating a good death, then we pave the way for compassion satisfaction.

When I see that dog resting on the table, peacefully, no longer fighting, no longer in pain, having died in the presence of her human family, I can see ethically indicated euthanasia for what it truly is: a gift.

## Data Availability

Not applicable.
